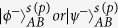# Complete hyperentangled-Bell-state analysis for photonic qubits assisted by a three-level Λ-type system

**DOI:** 10.1038/srep19497

**Published:** 2016-01-19

**Authors:** Tie-Jun Wang, Chuan Wang

**Affiliations:** 1 State Key Laboratory of Information Photonics and Optical Communications and School of Science, Beijing University of Posts and Telecommunications, Beijing 100876, China

## Abstract

Hyperentangled Bell-state analysis (HBSA) is an essential method in high-capacity quantum communication and quantum information processing. Here by replacing the two-qubit controlled-phase gate with the two-qubit SWAP gate, we propose a scheme to distinguish the 16 hyperentangled Bell states completely in both the polarization and the spatial-mode degrees of freedom (DOFs) of two-photon systems. The proposed scheme reduces the use of two-qubit interaction which is fragile and cumbersome, and only one auxiliary particle is required. Meanwhile, it reduces the requirement for initializing the auxiliary particle which works as a temporary quantum memory, and does not have to be actively controlled or measured. Moreover, the state of the auxiliary particle remains unchanged after the HBSA operation, and within the coherence time, the auxiliary particle can be repeatedly used in the next HBSA operation. Therefore, the engineering complexity of our HBSA operation is greatly simplified. Finally, we discuss the feasibility of our scheme with current technologies.

Entanglement plays an important role in quantum information processing (QIP)^1^. For example, entangled photons can act as the information carriers in quantum communication, such as quantum key distribution^2^,^3^ and quantum dense coding^4^,^5^. Also the entangled photons can serve as the quantum channel in quantum teleportation^6^, quantum secret sharing^7^,^8^,^9^ and quantum repeaters^10^ to connect the two neighboring nodes in a network as photons are regarded as the best flying qubits for QIP. The complete and deterministic analysis of the entangled Bell states is required in most fundamental quantum communication processes which exploit the nonlocal correlation of bipartite entanglement. Unfortunately, with only linear optical elements, a complete Bell-state analysis (BSA) is impossible and one can obtain the optimal success probability of 75% in identifying the four Bell states entangled in one degree of freedom (DOF) both in theory^11^ and in experiments^12^,^13^,^14^. In the past decades, much attention has been attracted on the BSA of photonic qubits^15^,^16^,^17^,^18^,^19^,^20^,^21^,^22^,^23^,^24^.

Hyperentanglement^25^,^26^,^27^,^28^ denotes the entanglement encoded on multi-DOF of a quantum system which is becoming an effective way to increase the channel capacity and to improve the performance of long-distance quantum communication. Recently, there are many presented works on hyperentanglement QIP, such as beating the channel capacity limit^28^,^29^,^30^,^31^,^32^, complete deterministic entanglement purification^33^,^34^, or assisting complete Bell-state analysis^26^,^27^,^35^. The complete distinguishing of the hyperentangled Bell states is required in most fundamental quantum communication processes which exploits the nonlocal correlation of hyperentanglement entanglement, such as establishing hyperentangled channel for superdense coding^29^ and the multi-DOF quantum teleportation for single photons^32^. In 2007, Wei *et al.*^36^ proved that with the help of linear optics, one can only distinguish 7 states out of the group of 16 orthogonal hyperentangled Bell states in two DOFs, and the upper bound of the maximal number of mutually distinguishable n-qubit Bell-like states is 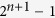, which is true for n = 1 and n = 2. In 2011, Lynn *et al.*^37^ provided a more general proof of this bound for Bell-state distinguishability. And if nonlinear optics is introduced, these 16 orthogonal Bell states can be distinguished completely^38^,^39^,^40^. In 2010, Sheng *et al.*^38^ proposed a scheme to distinguish the 16 hyperentangled Bell states completely with the help of cross-Kerr nonlinearity and discussed the application of this scheme in quantum communication. When a combined system composed of a single photon and a coherent probe beam passing through a cross-Kerr medium, a phase shift *θ* is picked up on the coherent probe beam. With the action of the cross-Kerr nonlinearity, one can distinguish the even-parity states from the odd-parity states in spatial-mode DOF of a two-photon system without destroying the two-photon system in the other DOF. Although the cross-Kerr nonlinearity in the optical single photon regime has been widely presumed, it remains quite controversial for the lack of experimental supporting with current techniques^41^,^42^.

The artificial atom and optical cavity coupled system is an essential platform for the realization of QIP. And the description of the system using cavity quantum electrodynamics (QED) plays an important role for information exchange between static and flying qubits in quantum communication networks and it has been demonstrated that, even in the bad-cavity regime, a measurable nonlinear phase shift between single photons can be achieved in a cavity QED system^43^. This nonlinearity can be realized by a variety of physical systems, such as a leaky resonator interacting with an atom or a quantum dot^44^,^45^,^46^. In 2010, Bonato *et al.*^46^ proposed the first proposal that uses interface between the photon and the spin of an electron confined in a quantum dot embedded in a microcavity operating for Bell-state analysis in the weak coupling regime. Also, a further incentive to study HBSA based on cavity coupling system lies in the recent advances of such systems^39^,^40^. In 2012, Ren *et al.*^39^ presented complete HBSA with the nonlinear optics based on a quantum dot(QD)-one-sided cavity system. In a one-sided cavity, due to the spin selection rule, the right circularly polarized light 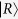 and the left circularly polarized light 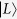 pick up two different phase shifts after being reflected from the QD-cavity system, and then, after two photons reflected by a cavity, the parity state of this photon pair in polarization DOF can be determined by measuring the state of the excess electron of the auxiliary QD without destroying the two-photon quantum system. However, in this work, there are four auxiliary QD-cavity coupled units which lead to in average 4 times two-qubit interactions between the photons and QDs, and the auxiliary QDs are all required to be prepared in a certain superposition spin state of the excess electron of the QDs and should be measured to read the parity information of the photon-pairs. In the same year, by using the double-sided QD-cavity system, Wang *et al.*^40^ presented a scheme for complete analysis of the hyperentangled Bell in both polarization and spatial-mode DOFs which requires only two auxiliary QD-cavity coupled units with 4 two-qubit interactions between the photons and QDs. In 2015, Liu *et al.*^47^ presented a scheme for the generation and analysis of hyperentanglement assisted by two nitrogen-vacancy (NV) centers in diamonds coupled with microtoroidal resonators. In these schemes, the nonlinearity between the photons and the auxiliary particles is used to construct the two-qubit controlled-phase operations which plays a critical role in HBSA protocols.

In this paper, we show that the complete differentiation of 16 hyperentangled Bell states in both polarization and spatial-mode DOFs for two-photon system can be efficiently achieved based on a two-qubit SWAP gate by using a three-level Λ-typle atom-cavity coupled unit interacting with single photons in reflection geometry. By replacing the usual two-qubit controlled-phase operations using the two-qubit SWAP gates, the interaction between the photons and the auxiliary particle is reduced to three times, and there is only one auxiliary particle required in our scheme. The initialization requirement of the auxiliary particle is reduced since it works as a temporary quantum memory and it is not required to be measured. Moreover, because the state of the auxiliary particle remains unchanged after the HBSA operation, within the decoherence time, the auxiliary particle can be repeatedly used in next HBSA operations. Compared with the previous HBSA schemes, the required experimental resource and the engineering complexity of the HBSA operation in our scheme is greatly simplified. And it is proved that the present scheme can both work in the weak- and strong-coupling regimes with current technologies. Finally, we discuss the feasibility of our scheme.

## Results

### The model of single-sided cavity and three-level Λ-type system

**Figure 1:**
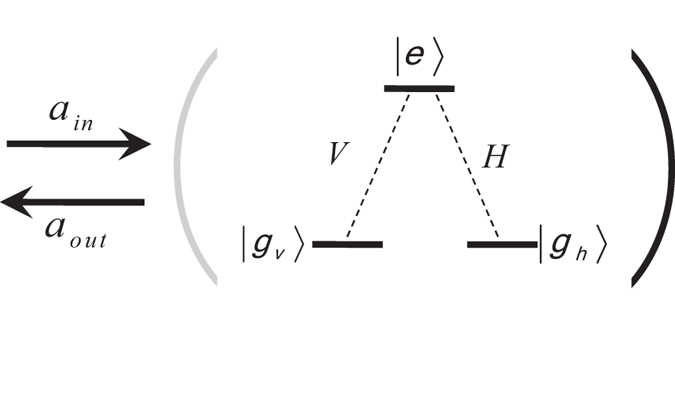
Interaction between a Λ system and a single photon propagating in one dimension. The Λ system is completely deexcited through radiative decay. The optically allowed transitions 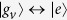 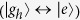 can only be excited by the single *V*-polarized (*H−*polarized) photon as the selection rules. Initially, the photonic and atomic qubits may be in arbitrary states. After reflection, the photonic and atomic qubits can be completely swapped under appropriate conditions.

Here we consider the case that an atom is trapped in a single-sided optical cavity, and the atom is assumed to be a three-level Λ-type system as shown in Fig. 1. The degenerate ground states of the atom, *i.e.*, 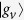 and 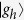, are considered to be the qubit states and the excited level 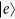 to be the ancillary state. The optically allowed transitions 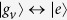 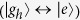 can only be excited by the single *V*-polarized (*H*-polarized) photon under the selection rules. The Hamiltonian 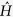 describes the interaction between the atom and the electric cavity field which is given by 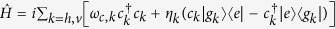, here we set 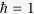. 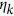 represents the light-matter interaction strength, 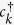 and 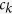 are the corresponding creation and annihilation operators for the k-polarization cavity field, respectively. The Hamiltonian 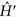 describes the interaction between the cavity field and the input-output fiber mode which is given by 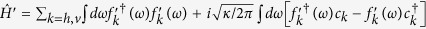. Here 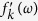 and 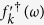 are the annihilation and creation operators for the k-polarized photon in the fiber mode, and 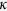 denotes the cavity-photon damping rate through the output mirror.

We assume that the atom is initially in state 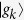 and the incoming pulse is in the k-polarization state at the beginning. By considering the spontaneous emission of the exited state 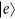 with the decay rate *γ*, the general time dependent wave function of the system can be described as^48^,^49^


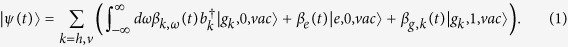


In the state 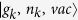, 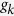 denotes the atomic state, *vac* describes the vacuum state in the fiber mode, and 0 or 1 means that the number of the photons in the k-polarization state. It is known that 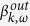, 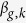 and 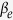 can be obtained by solving the Heisenberg equations of motion. In the rotating frame, the input-output relation are given by^50^,^51^


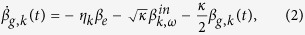



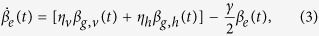



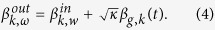


Here, 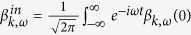 represents the input filed at the input-port of the one-sided cavity. 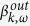 is the amplitude of the output pulse. By linearized the Eqs (2, 3, 4), we can get


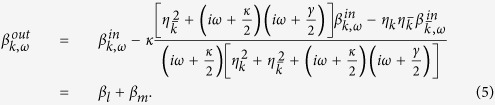


Here,


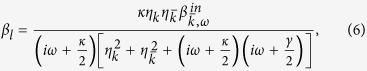



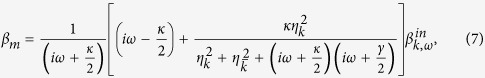


where 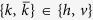 and 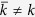. By taking 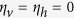, we get the the amplitude of the two output pulse 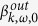 for an uncoupled cavity (or cold cavity) where the atom does not couple to the input filed,


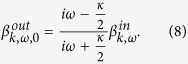


When 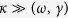, we have 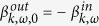. If the input photon is in resonance with the atom 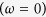, and the conditions that 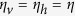 and 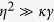 are satisfied, we have 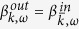 in Eq. (5).

Consider an ideal single-sided optical microcavity system (the side leakage and cavity loss can be neglected), when the atom is in the state 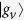, the *V*-polarized photon that interacts with the atom is reflected by the hot cavity, and turns the state of the atom into 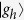. On the other hand, the *H*-polarized input photon could be reflected by the cavity with a *π* phase because of the resonance between the photon and the cold cavity. When the qubit is in the state 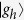, the *V*-polarized photon will be reflected by the cavity with a *π* phase while the input photon in the *H*-polarization will transfer the states of atom into 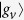. The evolution rule of the photon in different polarized states and different atomic states are described as


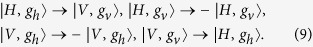


If the initial states of the photon and the atom are given by 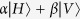 and 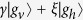, respectively, as illustrated in Eq. (9), the quantum states of atomic and photonic qubits are exchanged on reflection as 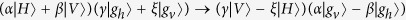. In the following, we use this system to implement complete hyperentangled-Bell-state analysis for the photonic systems.

### The hyper-SWAP gate using three-level Λ-cavity system

The optical properties of a three-level Λ-type atom in a single-sided microcavity can be used to construct a hyper-SWAP gate which can exchange the polarization qubit and the spatial-mode qubit between two photons. With this hyper-SWAP gate, one can distinguish the 16 hyperentangled-Bell-states in spatial-mode DOF and in polarization DOF for photonic systems.

**Figure 2:**
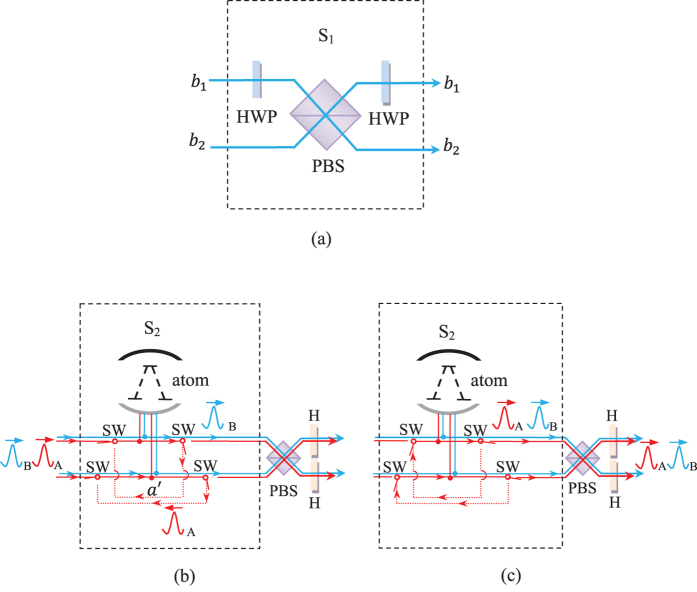
Schematic diagram for the hyper-SWAP gate using three-level Λ-cavity system. (**a**): Schematic diagram of the the 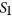 gate which can exchange the quantum polarization state and the spatial-mode state of the photon B with only the linear optical elements. The polarizing beam splitter (PBS) can transmit a horizontally polarized photon 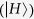 and reflect a vertically polarized photon 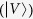. The half-wave plates (HWPs) with the angle of 45° to the horizontal direction can flip the polarization state of the photons 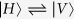. (**b**,**c**): Inside the dotted line, it is the schematic diagram of 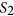 gate which can exchange the quantum polarization states between photon A and photon B using the atom-cavity coupled unit. SW represents an optical switch.

The framework of the hyper-SWAP gate circuit on the photonic qubits is shown in Fig. 2. Consider that the initial state of the atom is in the arbitrary state 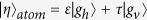, with 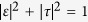. The photon pair AB is in an arbitrary state 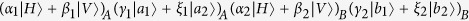, where 
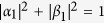
, 
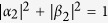
, 
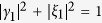
, and 
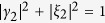
. The photons AB are in resonance with the atom, and they are forwarded to the atom with sufficiently large time intervals in sequence.

The detailed description of the hyper-SWAP gate operation between the polarization qubit of the photon A and the spatial-mode qubit of the photon B (shown in Fig. 2) could be described as follows:

**Step 1**: One should exchange the quantum polarization state and the spatial-mode state of the photon B using the linear optical elements (the 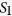 gate) shown in Fig. 2(a), that is


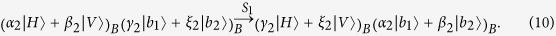


Here, the polarizing beam splitter (PBS) in Fig. 2(a) can transmit a horizontally polarized photon 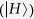 and reflect a vertically polarized photon 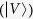. The half-wave plates (HWPs) in Fig. 2(a) with the angle of 45° to the horizontal direction can flip the polarization state of the photons 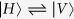.

**Step 2**: Simultaneously, the photon A is sent into the cavity to interact with the atom which is shown in the dotted line of Fig. 2(b),


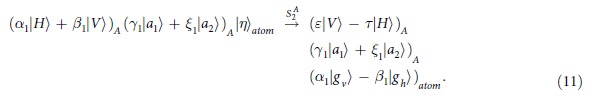


Here SW in Fig. 2(b) represents an optical switch. After photon A interacting with the atom, the SWs lead the photon A into the path 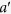 and the photon B is sent to the cavity to interact with the atom. Then the composite system of the atom and the photons AB will evolve as:


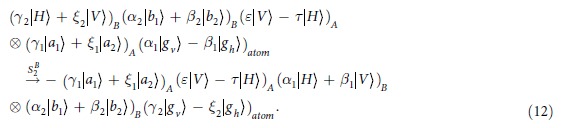


**Figure 3:**
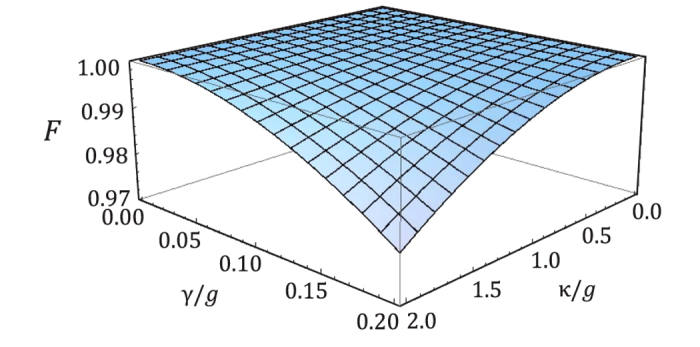
The fidelity (in amplitude) functions of *γ*/*g* and *κ*/*g* at g_*h*_ = *g*_*v*_ = *g* and *ω* = 0.

**Step 3**: Following the circuits shown in the dotted line of Fig. 3(c), by using the SWs, the photon A is recycled and interacted with the atom again. Finally, the composite state of the system evolves as:


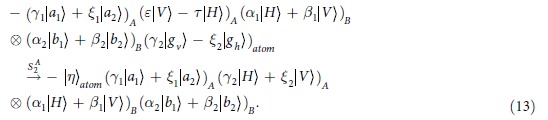


From Eq.(13), one can see that, by ignoring the overall phase of the photon-pair system, after the hyper-SWAP operation, the state of the atom remains unchanged. Meanwhile, the polarization information of photon A has be transferred into the the polarization DOF of the photon B, and the spatial-mode states of photon B can be transferred into the polarization DOF of photon A. In contrast, the polarization information of photon B has been transferred into the spatial-mode DOF of photon B, and the spatial-mode states of photon A remain unchanged.

### Complete HBSA using hyper-SWAP gate

A hyperentangled two-photon Bell state in both polarization and spatial-mode DOFs could be described as


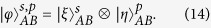


Here, the subscripts A and B represent the two photons in the hyperentangled state. The subscript s denotes the spatial-mode DOF, and 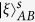 is one of the four Bell states in the spatial-mode DOF, which reads


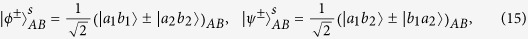


where 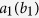 and 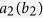 are the different spatial modes for the photon A(B). The subscript p denotes the polarization DOF, and 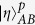 is one of the four Bell states in the polarization DOF, which are


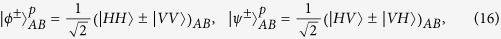


where H and V represent the horizontal and the vertical polarizations of photons, respectively.

Consider that the initial state of the atom is in an arbitrary state 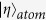, and the hyperentangled photon pair AB is in one of the 16 hyperentangled Bell states which is described as Eq. (14). By using the hyper-SWAP gate as shown in Fig. 2, the 16 hyperentangled-photon Bell states evolve as:


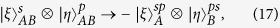


where 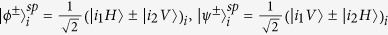, and 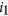
and 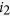 are the different spatial modes for the photon 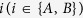. Then, as shown in Fig. 2(b,c), the photons pass through the PBS which can transmit the horizontal state and reflect the vertical one, and the *H* plate which is a half-wave plate with the angle of 22.5° to the horizontal direction can be used to implement the Hadamard operation in the polarization DOF [
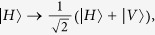
 
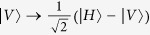
] on the photons. By ignoring the whole phase of the system, the two photons state evolves as


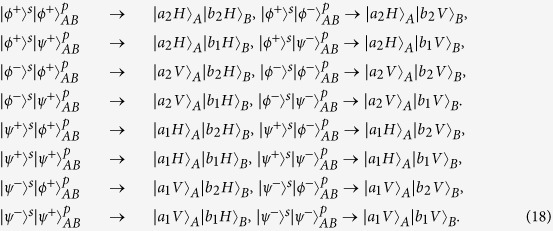


Then photons A and B can be measured independently in both the polarization and the spatial-mode DOFs with single-photon detectors, and the state of the atom remain unchanged. The relationship between the measurement outcomes of the final states of the two photons AB and the initial hyperentangled states of the two photons is shown in Table 1.

From Table 1, one can obtain the complete and deterministic analysis on quantum hyperentangled Bell states. The final state of the photon A(B) in spatial-mode DOF determines initial parity information in the spatial-mode(polarization) DOF of the hyperentangled two photons, whereas the outcomes of the photon A(B)’s polarization states determines the phase information in the spatial-mode(polarization) DOF of the hyperentangled two photons. In detail, when the photon A(B) is detected in 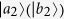 in the spatial-mode DOF, the photons AB are initially in the even-parity in the spatial-mode (polarization) DOF, that is 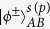. Meanwhile, if the final state of the photon A(B) is 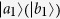 in the spatial-mode DOF, the photons AB are initially in the odd-parity in the spatial-mode (polarization) DOF, that is 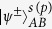. If the polarization state of the photon A(B) is detected in 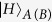, the initial phase information of the hyperentangled photons AB is “+” in the spatial-mode (polarization) DOF; otherwise, the initial phase information of the hyperentangled photons AB is “−” in the spatial-mode (polarization) DOF when the photon A(B) is detected in 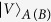. By far we have described the scheme of our HBSA using three-level is Λ-cavity system.

## Discussion

Compared with the previous scheme from refs 39,40,47, our scheme largely simplifies the HBSA operation process. For example, in Ren *et al.*‘s work^39^, there are four auxiliary QDs (for detecting the parity information and phase-information in polarization and spatial-mode DOFs of the hyperentangled photon-pair) and four auxiliary photons (for reading out the information encoded on the auxiliary QDs) are required for one time HBSA operation. In Liu *et al.*’s^47^ and Wang *et al.*’s^40^ schemes, there are two auxiliary artificial atomic qubits are required for one time HBSA operation, meanwhile, the auxiliary qubits must be prepared in a certain superposed spin state of the excess electron which will be measured after the HBSA operation to reveal the parity information of the photon-pairs. In our scheme, only one auxiliary particle is required for the 16 hyperentangled Bell states analysis, and the initial state of the auxiliary particle could be arbitrary of the ground states, pure or mixed. Moreover, within the coherent time, the auxiliary particle is not required to be measured and it can be repeatedly used in next HBSA operations. So the experimental resource and the engineering complexity of the HBSA operation in our scheme is greatly simplified. According to ref. 41, exploiting the one-sided cavity system which creates hyper-entanglement photon-pairs and the entanglement swapping operation, two remote nodes could be linked. Thus the present scheme exhibits potential applications in various physical systems for the long-distance quantum communication.

Moreover, the decoherence effect of the atom-photon system is also required to be considered. Once the auxiliary atom interacts with a photon, the coherence between the atom and the photon should be remained. At the same time, we emphasize that the initial state of the atom could be arbitrary of the ground states, pure or mixed, and it would not effect on the results of the hyperentangled Bell-state analysis. This statement is not contradictory to the importance of the coherence of the whole system composed of the atom and photons. That is because, no matter what the initial state of the atom is, pure (for example, 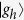 or 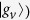 or mixed (for example, 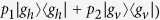, the auxiliary atom is just used as a temporary quantum memory, and after the hyper-SWAP operation(Step1–Step3), the atomic state is unchanged. During the whole process of our scheme, the coherence of the atomic state should be maintained, but the initial state of the atom can be prepared arbitrarily, pure or mixed of the ground states. This is an important difference between our scheme and other hyperentangled Bell-state analysis scheme in which the auxiliary particle must be prepared in the pure state and finally should be measured.

In summary, we proposed an efficient HBSA scheme for photonic system by replacing the usual two-qubit controlled-phase operations using the two-qubit SWAP gates. The interaction times between the photons and the auxiliary particle is reduced to three, and only one auxiliary particle is required in our scheme. The requirement of the auxiliary particle is reduced since it works as temporary quantum memories and need not to be actively controlled or measured. Moreover, as the state of the auxiliary particle remains unchanged after the HBSA operation, the auxiliary particle can be repeatedly used in the next HBSA operations within the coherence time. Therefore, the engineering complexity of the HBSA operation is greatly simplified compared with the previous HBSA scheme. Exploiting the existing experimental data, our calculation shows that this protocol are insensitive to both cavity decay and atomic spontaneous emission, so it can work in the case of a larger cavity decay rate, i.e., the cavity with a relatively lower-Q factor. All these advantages make this scheme more feasible in practical applications of long-distance quantum communication and scalable quantum computing.

## Methods

### Average fidelities and efficiencies of the gates

In this part, we give a brief discussion about the experimental implementation of our scheme. The level configuration under our consideration in Fig. 1 can be found in ^87^*Rb*; for example, the level with F = 1 (e.g., 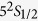 of Rb) acts as the ground state and the excited state could be 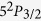. And 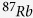 could be trapped at the center of an optical cavity^52^. By combining with the long trapping time of the atom in the cavity (typically, the atom trapping times are tens of seconds^53^), the atom can be considered as a good carrier of stationary qubits. We can calculate the fidelity and the efficiency in the case that the initial atomic state is 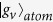, and the initial hyperentangled photon-pair (marked with A and B) is in the state 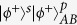. For simplicity, the input photons are assumed in resonance with the cavity. The fidelity of HBSA operation on the photon is F which could be described as


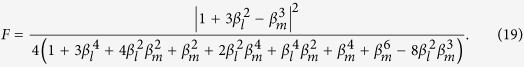


If the initial atomic state is 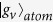 and the initial state of the hyperentangled photon-pair AB is 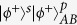, after 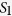 gate which performs the SWAP gate between the polarization and spatial-mode DOFs of the photon B, the state of the photon-pair AB becomes 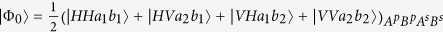, and then after the ideal hyper-SWAP gate, the state of the hybird system becomes 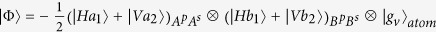. However, according to Eq.(5) in the unideal resonance case and after the hyper-SWAP gating opertion, the state of the system composed of photon-pair AB and the Λ-type atom becomes


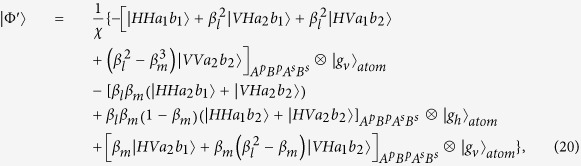


and so the fidelity of the hyper-SWAP gating opertion is 
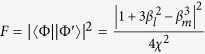
, here 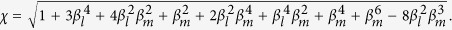


**Figure 4:**
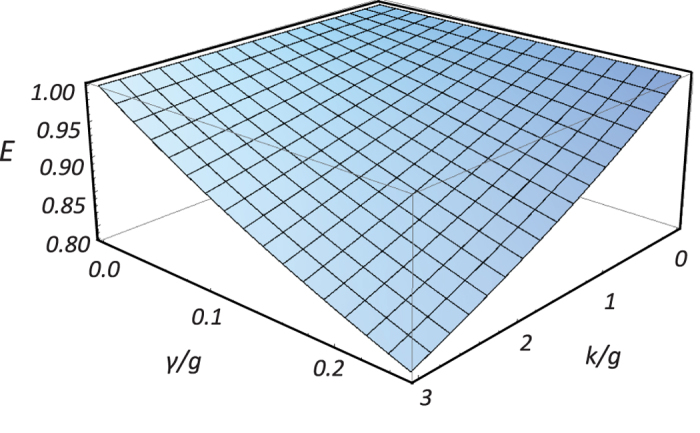
The efficiency (in amplitude) functions of *γ*/*g* and *κ*/*g* at *g*_*h*_ = *g*_*v*_ = *g* and *ω* = 0.

**Figure 5:**
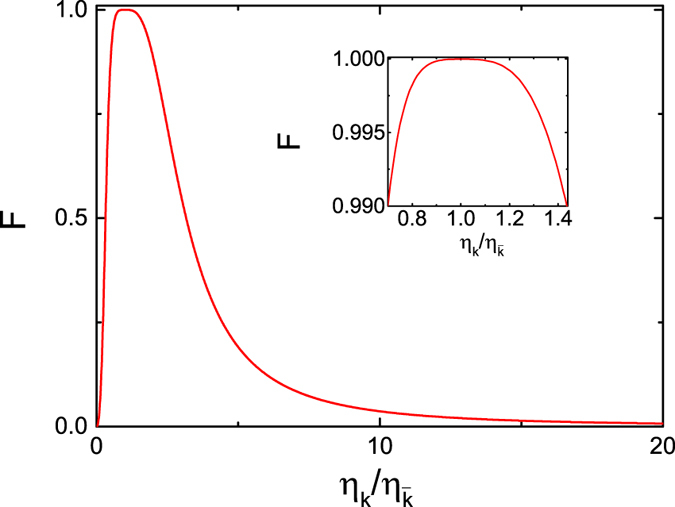
The fidelity (in amplitude) functions of the coupling strengths *g*_*h*_/*g*_*v*_ at *γ*/*g*_*v*_ = 0.1 and *κ*/*g*_*v*_ = 2.

The efficiency is given by 
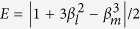
. The calculated results of the fidelity and the efficiency is shown in Figs 3 and 4. In Fig. 3, when 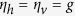, the atomic spontaneous emission *γ* and the cavity decay rate 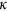 show a slight influence on the fidelity *F*. Even, it is of large decay rate, i.e., called bad cavity, the fidelity of the entangler gate is still above 0.98 where 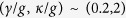. Using the values of the cavity-QED parameters 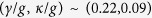
52,54, the gate fidelity is 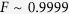. However, in the weak coupling regime, the efficiency reduces rapidly. From Fig. 4, the efficiency is below 0.8 where 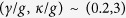. Figure 5 shows that the variance of the coupling strength 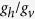 has a stronger influence on the fidelity. The attained fidelity is found to approach the ideal value when 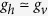. However, the fidelity is larger than 0.99 with 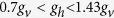. The present scheme can also be realized in other physical systems such as semiconductor quantum dots^55^, and superconducting system^56^ for the similar relevant levels. In general solid-state cavity-coupled system, the effective interaction between cavity-coupled qubits is described by the XY model or the Heisenberg exchange interaction. When a CPHASE gate or a CNOT gate is constructed using such interactions, generally, at least twice two-qubit interactions have to be invoked with complicated pulse sequences, but for a SWAP gate or iSWAP gate, only once two-qubit interaction is required^57^. Therefore, the development of SWAP-gate-based quantum algorithms would pave the way for an easier integration of solid-state qubits into a quantum communication network.

The challenge that two separate input light with different spatial-mode simultaneously interact with an auxiliary particle in cavity can be overcomed by using the optical switch at the single-photon level. One can switch multiple spatial modes into one light path with different time-bins, and so the single-sided cavity only interacts with a single spatial mode of light at different time intervals. Recently, the experimental studies of such system have attracted much attention, and we notice that the ultrafast all-optical switching by single photons^58^ has also been experimentally realized in QDCcavity system. Moreover, in ref. 59, the all-optical transistor which uses one photon to control the resonator transmission is also realized.

## Additional Information

**How to cite this article**: Wang, T.-J. and Wang, C. Complete hyperentangled-Bell-state analysis for photonic qubits assisted by a three-level Λ-type system. *Sci. Rep.* **6**, 19497; doi: 10.1038/srep19497 (2016).

## Figures and Tables

**Table 1:** Relation between the final states of the photon A(B) and the corresponding initial spatial-mode (polarization) states of the hyperentangled photon-pair AB.

The photon A(B)‘s final states	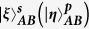
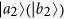	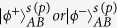
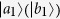	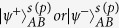
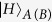	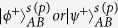
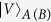